# A mild form of adenylosuccinate lyase deficiency in absence of typical brain MRI features diagnosed by whole exome sequencing

**DOI:** 10.1186/s13052-017-0383-7

**Published:** 2017-08-02

**Authors:** Marina Macchiaiolo, Sabina Barresi, Francesco Cecconi, Ginevra Zanni, Marcello Niceta, Emanuele Bellacchio, Giacomo Lazzarino, Angela Maria Amorini, Enrico Silvio Bertini, Salvatore Rizza, Benedetta Contardi, Marco Tartaglia, Andrea Bartuli

**Affiliations:** 1 Genetics and Rare Diseases, Research Division, Bambino Gesù Children’s Hospital, Piazza Sant’Onofrio 4, 00164 Rome, Italy; 2 Unit of Cell Stress and Survival Danish Cancer Society Research Center, Copenhagen, Denmark; 3 Institute of Biochemistry and Clinical Biochemistry, Catholic University of Rome, Rome, Italy; 4 Pharmacist Mother of a Patient affected by Adenylosuccinate lyase deficiency, Rome, Italy

**Keywords:** Adenylosuccinate lyase deficiency, Whole exome sequencing, Diagnosis, Epilepsy

## Abstract

**Background:**

Adenylosuccinate lyase (ADSL) deficiency is a defect of purine metabolism affecting purinosome assembly and reducing metabolite fluxes through purine de novo synthesis and purine nucleotide recycling pathways. The disorder shows a wide spectrum of symptoms from slowly to rapidly progressing forms. The most severe form is characterized by neonatal encephalopathy, absence of spontaneous movement, respiratory failure, intractable seizures, and early death within the first weeks of life. More commonly, ADSL presents purely neurologic clinical picture characterized by severe psychomotor retardation, microcephaly, early onset of seizures, and autistic features (type I) or a more slowly progressing form with later onset, and major features including slight to moderate psychomotor retardation, and transient contact disturbances (type II). Diagnostic markers are the presence of succinylaminoimidazole carboxamide riboside (SAICAr) and succinyladenosine (SAdo) in extracellular fluids. ADSL is a rare disorder, although its prevalence remains unknown. Of note, the wide range of essentially nonspecific manifestations and lack of awareness of the condition often prevent diagnosis.

**Case presentation:**

We present here the case of particularly mild, late onset ADSL that has been unsuccessfully investigated until whole exome sequencing (WES) was performed.

**Conclusions:**

Besides emphasizing the valuable diagnostic value of WES, this report provides new data further documenting the relatively wide clinical manifestation of ADSL.

**Electronic supplementary material:**

The online version of this article (doi:10.1186/s13052-017-0383-7) contains supplementary material, which is available to authorized users.

## Background

Adenylosuccinate lyase deficiency (ADSL, OMIM #103050) is an autosomal recessive defect of purine metabolism, resulting from biallelic inactivating mutations in the *ADSL* gene, and associated with a wide range of clinical manifestations. The disease was first described more than 30 years ago by Jaeken and van den Berghe, in three patients with severe psychomotor delay, autistic features, and succinylpurines in the cerebrospinal fluid (CSF), plasma and urine [1]. The ADSL enzyme is involved in two pathways of purine nucleotide metabolism, i.e., the conversion of succinyl-aminoimidazole carboxamide riboside (SAICAr) into aminoimidazole carboxamide ribotide (AICAr) along the de novo pathway, and formation of adenosine monophosphate (AMP) from adenylosuccinate (S-AMP). ADSL deficiency results in marked elevation of succinyladenosine and SAICAr in various bodily fluids, particularly in CSF and urine.

ADSL deficiency is characterized by marked clinical variability, ranging from a fatal neonatal form to milder conditions with infancy onset. The fatal form is characterized by neonatal encephalopathy, absence of spontaneous movement, respiratory failure, and intractable seizures, resulting in early death within the first weeks of life [2]. A relatively milder but still severe phenotype includes severe psychomotor retardation, microcephaly, early onset of seizures, and autistic features (type I). A more slowly progressing form has also been described (type II), as having later onset, usually within the first years of life, slight to moderate psychomotor retardation, and transient contact disturbances, epilepsy, and visual impairment [3]. The disease manifests symptoms along a continuum and, despite the utility of communicating with the above mentioned three descriptive categories, there are no fixed parameters to ascribe a particular patient to a single category. In principle, diagnosis could be possible by determining succinyladenosine and SAICAr in urine; the wide range of essentially nonspecific manifestations and lack of awareness of the condition, however, generally prevent a prompt and correct diagnosis, with direct impact on patient management and counseling.

Here, we present the case of a mild, late onset ADSL with no obvious signs of disease progression and degradation, without “more classical signs” like visual impairment, hypomyelination or microcephaly that has been largely and unsuccessfully investigated until diagnosis was reached by whole exome sequencing (WES).

## Case presentation

**Fig. 1:**
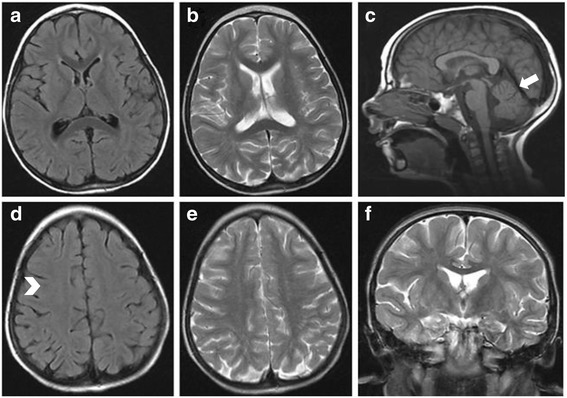
MRI of the patient at age 4 years. The images show very mild abnormalities of the white matter that are barely visible in FLAIR (**a, d**) (*arrow head*). Sagittal T1 weighted image shows a normal corpus callosum and a mild increased spacing of the cerebellar folia (*arrow*) (**c**). Coronal and axial T2 weighted images document peculiar fine stripes in T2 weighted image (**b**, **e**, **f**)

A girl of 9 years presented with a history of global development delay and epilepsy. First of two siblings of unrelated healthy parents, the patient was born at 41 weeks of gestational age after an uneventful pregnancy. The neonatal period was unremarkable with growth parameters in the normal range. Developmental milestones were globally delayed: she was sitting independently at the age of 9 months, started to walk independently at 30 months with an ataxic and uncertain gate, but she walked and ran normally at the age of 4 years. She showed severe language delay and, at the age of 9, pronounced around 20 words. She presented with seizures at 3 years and 9 months that ranged from partial seizures, myoclonus to generalized seizures only partially controlled by drugs. At 4 years, brain magnetic resonance imaging (MRI) revealed very mild abnormalities of the white matter and a mega cisterna magna. The corpus callosum appeared normal, and a mild increased spacing of the cerebellar folia was noted (Fig. 1).

The child had been evaluated at many different third level centers and a number of metabolic and genetic tests had been performed, including standard karyotype, array-CGH, *SHANK3* mutation scan, and very long chain fatty acid, transferrin isoform, plasmatic amino acids and urinary organic acid analyses. All laboratory tests provided negative results.

At the age of 9 years, the girl presented with a global developmental delay (her mental age at9, was 4 years), slight autistic feature (stereotypies such as hand movements, repetitive manipulation of toys, clapping hands, rubbing feet, and stereotyped sounds), language delay, myoclonic seizure only partially controlled by drugs (sodium valproate in association with clobazam). In Table 1 the main clinical and imaging features of ADSL compared with patient are reported.

**Table 1:** Main clinical and imaging features of ADSL and of the patient

Main clinical and imaging features of ADSL	Patient features
- Growth retardation	-
Head and Face	
- Brachycephaly	-
- Microcephaly	-
- Prominent metopic suture	-
- Thin upper lip	-
- Long smooth philtrum	-
- Low-set ears	-
- Strabismus	-
- Nystagmus	-
Neurologic	
Central Nervous System	
- Psychomotor delay,	+
- Mental retardation	Mild
- Hypotonia	-
- Gait ataxia	First two years of life
- Inability to walk	-
- Poor eye contact	+
- Poor language and speech development	+ partially controlled
- Seizures	-
- Spasticity	-
- Opisthotonus	-
- Myoclonus	-
- Brisk reflexes	-
- Cerebral atrophy	-
- Cerebellar atrophy	-
- Hypomyelination	-
- Atrophy of corpus callosum	-
	-
Behavioral Psychiatric Manifestations	
- Autistic features	+/−
- Hyperactivity	-
- Aggressive behavior	-
- Temper tantrums	-
- Stereotypic movements	+
- Self-mutilation	-
- Happy demeanor	+
- Inappropriate laughter	-

After a prior workup no additional targeted test were considered useful for the etiological evaluation, so the need for WES was considered.

## Methods

### Whole exome sequencing

Informed and written consent from all family was obtained. Targeted enrichment and massively parallel sequencing were performed on genomic DNA extracted from circulating leukocytes of the proband and her parents. Exome capture was carried out using SureSelect Human All Exon V.4 (Agilent). Sequencing data analysis was performed using an in-house implemented pipeline which mainly takes advantage of the Genome Analysis Toolkit (GATK V.3.4) framework [4], as previously reported in detail [5–7]. WES statistics and data analysis are provided in Additional file 1: Table S1.

### Genetic analysis

Sequence validation and family segregation analyses were performed by Sanger sequencing.

### Assay of ADSL enzymatic activity and HPLC analyses

Packed erythrocytes were treated to obtain a 20% haemolisate, and 5 ml were incubated for 20 min at 37 °C in presence of ADS 250 mM (Sigma- Aldrich), in 400 ml of a proper incubation buffer. ADSL activity was determined using a time-course assay, and calculated on the basis of the total amount of AMP produced in the reaction mixture during incubation, with respect to the value of this compound determined in control erythrocytes. Samples obtained at different times, were deproteinized as previously reported [8], and 100 ml of supernatant were utilized for the AMP analysis, performed by an ion-pairing HPLC method, allowing the synchronous separation of several compounds for the chemical diagnosis and screening of inborn errors of metabolism [9]. The same HPLC method was utilized for the S-Ado and SAICAr determination in urine sample of the patient.

## Results

### WES analysis

**Fig. 2:**
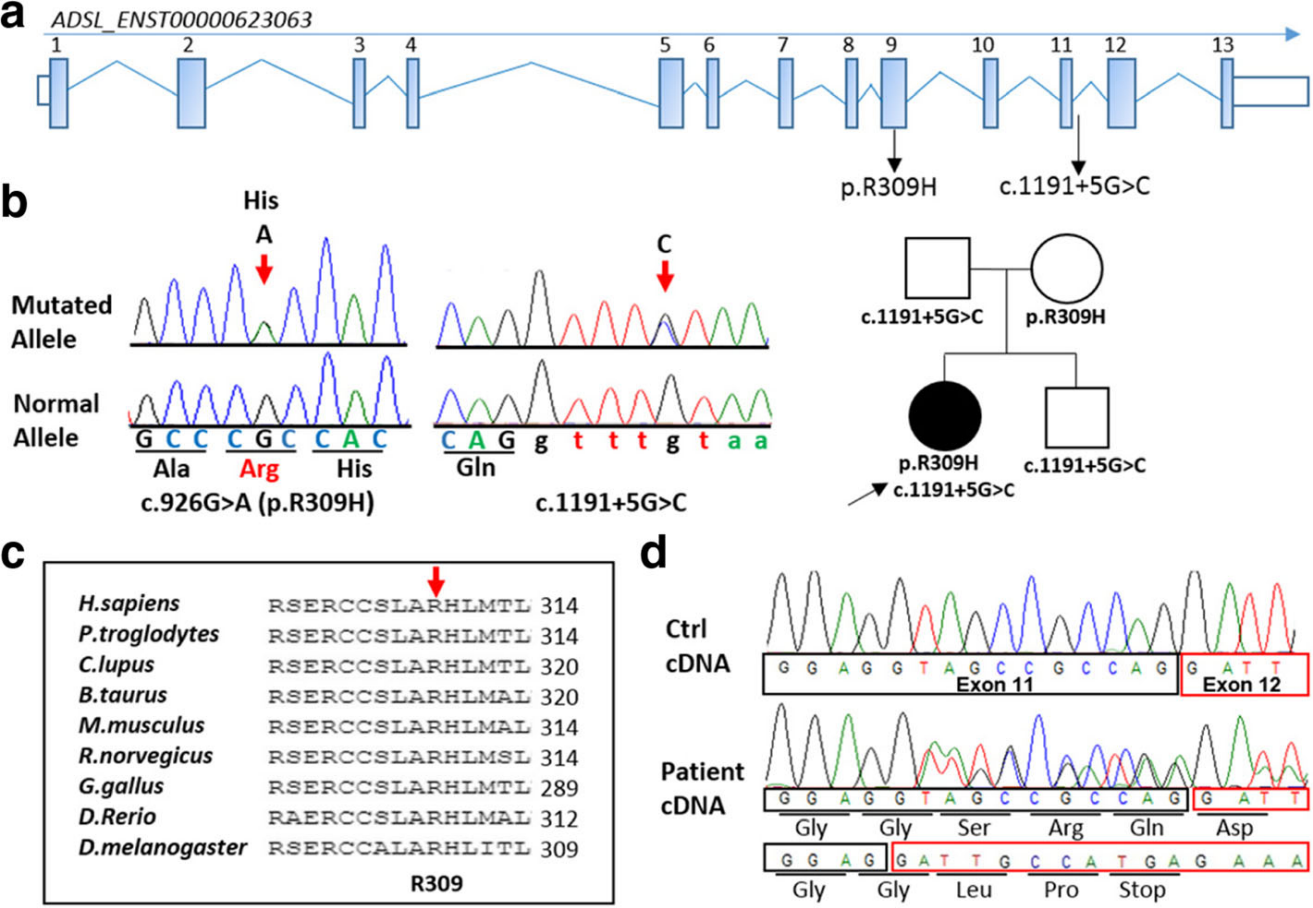
Molecular characterization of ADSL mutations. **a** Schematic representation of the *ADSL* transcript (ENST0000062306) with positions of the two mutations identified by WES. **b** Electropherograms showing the missense c.926G > A, p.R309H, and splice site c.1191 + 5G > C mutations (indicated with *red arrows*) occurring in trans in the proband. On the right side of the panel the family tree and segregation are shown.**c** Multiple sequence alignment of the human ADSL sequence of the region encompassing the disease-associated amino acid change (*red arrow*) with its orthologues. Arg309 is evolutionarily conserved among vertebrates. **d** Schematic representation of proper and aberrant splicing of *ADSL* exons 11 (*black box*) and 12 (*red box*). In the presence of the c.1191 + 5G > C change, the normal splice donor site is no longer recognized and an alternative cryptic splice donor site is used instead, resulting in loss of the distal sequence of exon 11, and in a frameshift causing a premature stop of translation

Among a total 57,433 called variants, data annotation predicted 12,363 high-quality variants having functional impact (i.e., non-synonymous, indels and splice site changes) (Supporting information, Additional file 1: Table S1). Among them, 310 private and rare changes were retained for further analyses. Only changes predicted to be deleterious by CADD v.1.3 (score > 15.0) and/or dbNSFP SVM v.2.8 (radial score > 0.0) algorithms were retained. Variants were prioritized on the basis of the functional relevance of genes, taking into account X-linked, autosomal dominant and autosomal recessive inheritance models (Additional file 1: Table S1). Variant filtering and prioritization allowed to identify two compound heterozygous variants in the *ADSL* gene as the only excellent candidates as causative event underlying the trait (Fig. 2a). Both variants were validated by Sanger sequencing and confirmed to be inherited in trans (Fig. 2b). One variant was missense, p.Arg309His (c.926 G > A), and affected a highly conserved arginine residue (Fig. 2c), while the other was a splice site change c.1191 + 5G > C inherited from the father and present also in the healthy brother. To verify the impact of the splice site change on transcript processing, RNA sequencing was performed, which confirmed aberrant processing of the ADSL transcript. Specifically, the mutated mRNA was 11-nucleotide shorter than the wild-type, and the missing nucleotide stretch corresponded to the distal sequence of exon 11 (Fig. 2d). Exon 12 was found to be spliced on to the remaining exon 11, at position c.1180, due to the use of a cryptic splice donor site. Translation of this aberrant transcript predictsan ADSL protein with a premature stop codon (Fig. 2d).

### Structural analyses

**Fig. 3:**
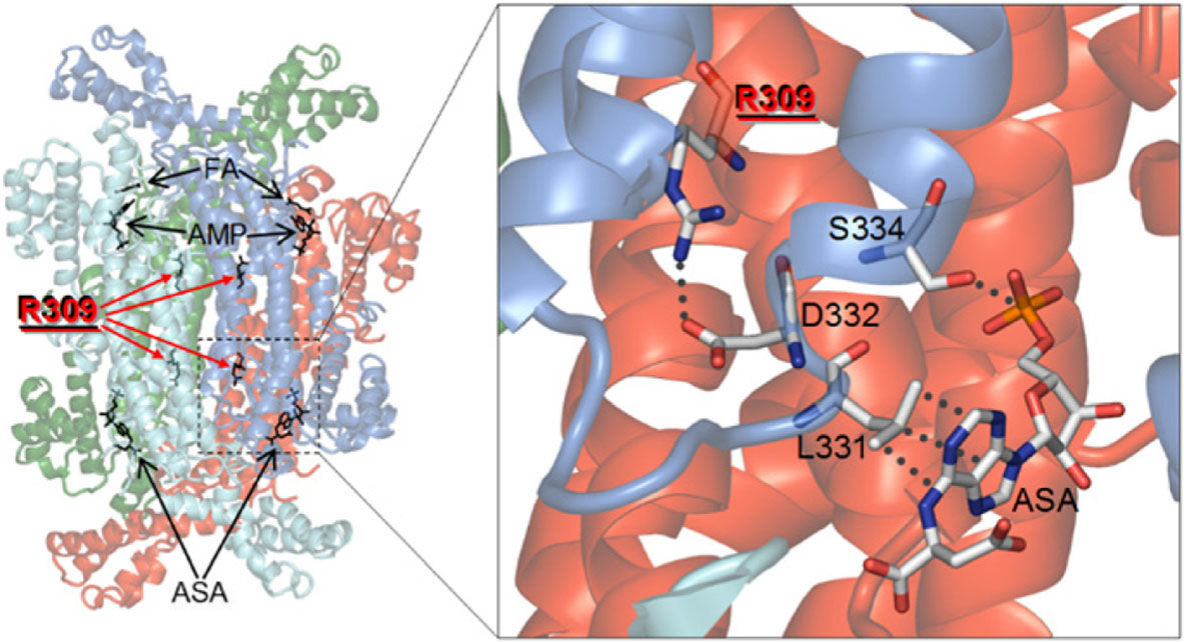
Three-dimensional mapping of the Arg309His substitution in the ADSL holoenzyme. On the left side of the panel, localization of Arg^309^ in the ADSL protein homotetramer (the four subunits are in different colors) complexed with adenylosuccinic acid (S-AMP) substrate, and its enzymatic products adenosine monophosphate (AMP) and fumaric acid (FA) (PDB accession number 2VD6). On the right side of the panel, the inset shows a slab view of Arg^309^ in one ADSL subunit forming a salt-bridge with Asp^332^ of the neighboring subunit, and the nearby Leu^331^ and Ser^334^ that bind to the substrate (interactions are represented by dots between atoms)

To explore the impact of the p.Arg309His change on ADSL function, inspection of the structural consequences of the amino acid substitution on the holoenzyme organization was obtained. Arg^309^ is located at the interface between subunits in the functional ADSL homotetramer contributing to holoenzyme stabilization by forming a salt-bridge with Asp^332^ in a region close to the active sites of the enzyme (Fig. 3). Due to symmetry reasons, four identical interactions occur in the homotetramer contributing to its four equivalent catalytic regions. Of note, this substitution occurs in a region of densely packed residues (Fig. 3). Through the salt-bridge, with residue Asp^332^, Arg^309^ also contributes to stabilize the conformation of residues flanking the Asp^332^ side chain with the bulk and rigid imidazole ring, with possible impact of the conformation of the substrate binding region. Overall, these analyses predicted an effect of the amino acid substitution on the overall structure of the holoenzyme and its catalytic function.

### Biochemical analyses

To test our in silico structural prediction, the ADSL catalytic activity was analyzed in vitro, and found to be significantly reduced in erythrocytes (80.95 IU/I), corresponding to approx. 25% of the lower value of normal range of healthy control subjects (320–450 IU/l erythrocytes). Contextually, concentration of urinary purine metabolites revealed a high elevation of S-Ado (115.45 mmol/mmol creatinine) and SAICAr (57.59 mmol/mmol creatinine) which are not detectable in control samples.

## Discussion and conclusion

ADSL deficiency is a rare autosomal recessive progressive disorder with central nervous system involvement. The disease manifests symptoms along a continuum ranging from a neonatal lethal condition to a milder slowing progressing form generally manifesting in the first years of life. Clinical heterogeneity is especially prominent in the less severe end of the spectrum, and some patients with an “attenuated phenotype” may present no obvious signs of disease progression. Incidence of ADSL deficiency remains unknown. The wide clinical spectrum of the disease and absence of pathognomonic features account for difficulties in ADSL diagnosis and differential diagnosis.

We report about an atypical case of ADSL deficiency, which has largely and unsuccessfully been investigated (three University Centers and a pediatric multispecialistic Hospital) until WES was performed. The patient presented with a quite unspecific clinical phenotype, in which the relatively mild form of the condition was characterized by the absence of some major characteristic features of ADSL deficiency, including visual impairment, microcephaly and hypomyelination [10].

When the disease is suspected, and the clinical picture is suggestive, clinical diagnosis of ADSL deficiency can be easily confirmed by urinary/plasma screen and/or *ADSL* mutation analysis. However, purine urinary screen is not widely available and, moreover, with ADSL being a rare and clinically variable disease, diagnosis based on clinical assessment can be challenging. In the case described, the diagnosis was reached through a reverse pathway (from molecular to biochemical confirmation), after a long period of diagnostic uncertainty. This report enlightens the power of WES as first-line tool, to obtain diagnosis of complex tracts or “orphan” disorder.

In the absence of any gestaltic approach, the genetic heterogeneity of many Mendelian neurologic/metabolic disorders and variable clinical impact of mutations in individual genes often represent obstacles to the use of “phenotype”-driven genetic testing. Even with the availability of chromosomal microarray testing and more disease-specific genetic panels, prior to WES, the diagnostic rate in pediatric neurodevelopmental disorders has remained around 25%. The introduction of WES in the diagnostic workflow has significantly improved the diagnostic rate in pediatric neurology patients, up to 48% in some specific patient cohort [11]. Moreover, stepwise diagnostic testing based on the use of single gene or gene panels in the evaluation of pediatric neurology patients is time consuming and costly, as in the specific case reported, placing the additional burden of prolonged diagnostic uncertainty on families. Besides the high diagnostic yield, WES also is emerging as an economically convenient diagnostic tool if performed early in the diagnostic evaluation. A recent paper estimated that with the use of WES there is the potential for an estimated average savings of $2465.62 and $502.52 (the average charge estimate on secondary genetic and metabolic testing, respectively) and up to $13,305.00 and $2340.00 (the maximum charge estimate on secondary genetic and metabolic testing, respectively). It is suggested that WES charge could be lower than the composite required for more conventional secondary genetic and metabolic laboratory testing [12]. Limiting the molecular screening to the Mendeliome, which comprehends the genes that are known to cause a Mendelian disorder, WES appears a highly informative and convenient first-tier tool in the diagnostic setting. Of note, in unsolved cases, WES reanalysis can be expected to provide diagnosis since the constant improvements in WES data analysis and unceasing identification of new disease genes. Moreover, by providing a full profile of the extent of variation contained in the protein-coding portion of the genome, WES data reanalysis in a research-oriented environment is expected to allow the identification of potential candidate genes in a significant proportion of cases. Investigating causality of the sequence variants in human disease becomes an important part in NGS for the research and clinical applications. Recently, important guidelines on them have been published and will keep on updating. [13]. Implementing this approach in the clinical setting, however, requires tight collaboration between clinicians, geneticists and molecular biologists since the concerted effort required for a correct interpretation and validation of the functional impact and clinical relevance of the identified sequence variants.

Increasing the diagnostic rate has high clinical relevance even if prospects for treatment or disease-altering management are not prominent, like in ADSL. Diagnosis helps the patient and family members gain access to appropriate patient management as well as to organizations that provide emotional support as well as practical advice. Furthermore, accurate diagnosis is vital so that families can receive genetic counseling about risk recurrence. WES can significantly improves the diagnostic rate in pediatric neurology patients and likely will become a valid first tier evaluation for a wide variety of neurologic disorders with nonspecific or complex phenotypes, contributing to the identification of new mutations and a delineation of the clinical variability characterizing disorder.

Despite the increasing number of ADSL-deficient patients reported, there are only a few communications of therapeutic considerations and efforts. Two papers showed some beneficial effects (D-ribose and uridine administration) [14, 15]. D-ribose administration, which increases the provision of phosporibosylpyrophosphate (PRPP) and stimulates de novo purine synthesis, has been applied in a few ADSL patients. Although promising results in motor coordination and seizure control in a 13-year-old female after several months of D-ribose therapy [16] they have not been confirmed in further studies.

In 2013 Werkhoven et al. evaluated S-adenosyl-l-methionine (SAMe) as a potential treatment for ADSL deficiency [17] but there was no clear response evidenced in urine metabolite levels or clinical. Reports about positive use of a ketogenic diet for treatment of refractory epilepsy have also been reported [18]. None of the mention treatments has been used for the patient.

## Additional file

Additional file 1: Table S1. Whole exome sequencing data output. (DOCX 12 kb)

## Abbreviations

ADSL: Adenylosuccinate lyase deficiency
AICAr: Aminoimidazole carboxamide ribotide (AICAr)
AMP: Adenosine monophosphate
CSF: Cerebrospinal fluid
MRI: Rain magnetic resonance imaging
SAdo: Succinyladenosine
SAICAr: Succinylaminoimidazole carboxamide riboside
S-AMP: Adenylosuccinate
WES: Whole exome sequencing
