# Supplementary material for: A mild form of adenylosuccinate lyase deficiency in absence of typical brain MRI features diagnosed by whole exome sequencing
Source: Ital J Pediatr. 2017 Aug 2;43:65. doi: 10.1186/s13052-017-0383-7 (PMC5541734; doi:10.1186/s13052-017-0383-7)
Supplement: Additional file 1: Table S1. — Whole exome sequencing data output. (DOCX 12 kb) [file 13052_2017_383_MOESM1_ESM.docx]

**Table S1.** Whole exome sequencing data output.

| Target region coverage^1^ | 99.8% |
| --- | --- |
| Target region coverage, 20x^1^ | 94.4% |
| Average depth on target | 82x |
| Coverage of target region | 99.8% |
| Average read length | 90 bp |
| Total number of variants | 57.433 |
| Number of variants with predicted functional effect | 12,363 |
| Novel, clinically associated, and unknown/low frequency variants^2^ | 381 |
| Putative disease genes (autosomal dominant trait)^3,4^  Filtered candidate genes^5^ | 1  0 |
| Putative disease genes (autosomal recessive trait)^6^  Filtered candidate genes^5^ | 1, *ADSL*  1 |
| Putative disease genes (X-linked trait)  Filtered candidate genes^5^ | 0  0 |

^1^Referred to SureSelect Human All Exon V.4 (Agilent).

^2^MAF <0.1% in dbSNP142 and ExAC V. 0.3 databases, and with frequency <2% in our *in-house* database.

^3^Only *de novo* changes are considered, as both parents are unaffected.

^4^ *ATP5SL* (c.565G>A, G189S, NM_001167867.1, rs367830809).

^5^Filtering retained functionally relevant variants (*i.e.*, nonsynonymous and splice site changes, excluding variants predicted as benign by CADD and metaSVM algorithms).

^6^ *ADSL* (c.1191+5G>C; c.926G>A, p.R309H);
